# *Lactobacillus plantarum*-Derived Postbiotics Ameliorate Acute Alcohol-Induced Liver Injury by Protecting Cells from Oxidative Damage, Improving Lipid Metabolism, and Regulating Intestinal Microbiota

**DOI:** 10.3390/nu15040845

**Published:** 2023-02-07

**Authors:** Wei Ye, Zengqiang Chen, Zhuoqi He, Haochen Gong, Jin Zhang, Jiaju Sun, Shanshan Yuan, Junjie Deng, Yanlong Liu, Aibing Zeng

**Affiliations:** 1 School of Laboratory Medicine and Life Science, Wenzhou Medical University, Wenzhou 325035, China; 2 Healthcare Center of the First Affiliated Hospital of Wenzhou Medical University, Wenzhou 325000, China; 3 Wenzhou Institute, University of Chinese Academy Sciences, Wenzhou 325000, China; 4 School of Pharmaceutical Science, Wenzhou Medical University, Wenzhou 325035, China

**Keywords:** *Lactobacillus plantarum*, postbiotic, intestinal microbiota, alcoholic liver injury

## Abstract

Here, the aim was to evaluate the protective effect of *Lactobacillus plantarum*-derived postbiotics, i.e., LP-cs, on acute alcoholic liver injury (ALI). After preincubation with LP-cs, HL7702 human hepatocytes were treated with alcohol, and then the cell survival rate was measured. C57BL/6 male mice were presupplemented with or without LP-cs and LP-cs-loaded calcium alginate hydrogel (LP-cs-Gel) for 3 weeks and given 50% alcohol gavage to establish the mouse model of ALI, LP-cs presupplementation, and LP-cs-Gel presupplementation. The histomorphology of the liver and intestines; the levels of serum AST, ALT, lipid, and SOD activity; liver transcriptomics; and the metagenome of intestinal microbiota were detected in all mouse models. In vitro, LP-cs significantly increased the survival rate of alcohol-treated cells. In vivo, presupplementation with LP-cs and LP-cs-Gel restored the levels of serum AST, ALT, and SOD activity, as well as TC and TG, after acute alcohol intake. In the LP-cs-presupplemented mice, the genes involved in fatty acid metabolic processes were upregulated and the genes involved in steroid biosynthesis were downregulated significantly as compared with the ALI mice. LP-cs significantly increased the abundance of intestinal microbiota, especially *Akkermansia muciniphila*. In conclusion, LP-cs ameliorates ALI by protecting hepatocytes against oxidative damage, thereby, improving lipid metabolism and regulating the intestinal microbiota. The effect of LP-cs-Gel is similar to that of LP-cs.

## 1. Introduction

Excessive alcohol consumption is the primary cause of alcoholic liver disease (ALD), including hepatic steatosis, steatohepatitis, fibrosis, cirrhosis, and potential hepatocellular carcinoma [1]. Although alcohol withdrawal can alleviate alcoholic liver injury (ALI), it cannot completely reverse liver injury that has been caused by excessive drinking. Currently, there is no effective drug treatment to prevent the development of ALD in long-term alcoholics or in subjects with acute excessive alcohol consumption.

The intestinal microbiota are considered to be a virtual metabolic organ that is connected with extraintestinal organs by making various axes. The gut–liver axis, which promotes the interaction of metabolism, immunity, and the neuroendocrine system between the intestines and liver, is beneficial to the regulation and stabilization of symbiosis between the intestinal microbiota and liver [2]. Alcohol has been shown to disturb the structure and composition of the intestinal microbiota, leading to impairment of intestinal integrity and liver function. Therefore, intestinal microbiota are considered to be a potential target for the treatment of ALD [3].

Probiotics and their metabolites have been indicated to regulate the composition of intestinal microorganisms directly or indirectly under alcohol exposure and have anti-inflammatory and antioxidant capacities; they can regulate lipid metabolism in the liver, thus, effectively maintaining the integrity of the intestinal barrier and reducing liver damage [2]. Currently, the most commonly used probiotics are *Lactobacillus* spp. and *Bifidobacteria* spp., which are lactic acid bacteria (LAB). The beneficial effects of *Lactobacillus rhamnosus*, *Lactobacillus casei*, *Lactobacillus fermentum*, *Lactobacillus plantarum*, and *Bifidobacterium longum* used alone or in combination and their fermented products on ALI have been reported in previous studies [4,5,6,7,8]. Among them, *Lactobacillus rhamnosus* and *Lactobacillus plantarum* are the most widely used strains.

In earlier studies, the protective effects of *Lactobacillus rhamnosus* GG (LGG) and its culture supernatant (LGGs) in ALI have received more attention. It has been demonstrated that LGG and LGGs were beneficial for the regulation of intestinal microbiota, as well as improvement of intestinal immunity and intestinal barrier function in ALI [9,10,11,12]. In recent studies, various strains of *Lactobacillus plantarum* were also applied to maintain intestinal barriers and protect against alcohol-induced liver injury. These strains were shown to improve intestinal epithelial dysfunction, protect the intestinal barrier [8,13], regulate the intestinal microbiota [14,15,16], and had antioxidant and anti-inflammatory properties for balancing liver lipid levels [8,13,14,15,16,17,18]. Moreover, in these studies, the potential molecular mechanisms were investigated, involving the Nrf2 [18] and MAPK signaling pathways [16].

Recent studies have also noted that a range of cell structures and metabolites produced by LAB fermentation were beneficial to the host, such as various cell components, short-chain fatty acids (SCFAs), lactic acid, and bioactive peptides [19]. These effector molecules are mainly derived from inanimate microorganisms, including their components or end-products. Therefore, the concept of postbiotics was proposed. This newly defined concept belongs to the family of probiotics, prebiotics, and synbiotics. A postbiotic is defined as a preparation of inanimate microorganisms and/or their components that confers a health benefit to humans [20]. It is a complex mixture that includes the components of inanimate microorganisms, enzymes, proteins, peptides, SCFAs, organic acids, and vitamins. As compared with probiotics, postbiotics are safer, more stable, and easier to quantify. Due to these advantages and the benefits for host health, postbiotics have attracted more attention in recent years.

The current study focuses on the protective effects of *Lactobacillus plantarum* AB161 culture supernatant (LP-cs), a *Lactobacillus plantarum*-derived postbiotic, on alcohol-induced liver injury. This strain of *Lactobacillus plantarum* was isolated from the white sediment of naturally fermented wine. In previous studies, *Lactobacillus plantarum* AB161 showed alcohol resistance, antioxidative capacity, and hydrolysis of bile acid. Based on previous research findings on the role of *Lactobacillus plantarum* in ALI treatment, we speculated that the effects of LP-cs might be similar to those of *Lactobacillus plantarum*, which have been less discussed. Therefore, in this study, the hepatocytes and the experimental mice were presupplemented with LP-cs, and then treated with a high dose of alcohol in a short time to establish the models of acute ALI. Subsequently, the effects of LP-CS on the models were evaluated in vivo and in vitro. Additionally, considering the manner of administration, LP-cs-loaded calcium alginate hydrogels (LP-cs-Gel) were administrated to the experimental mice to evaluate the effects on ALI.

## 2. Materials and Methods

### 2.1. Preparation and Administration of Lactobacillus plantarum

*Lactobacillus plantarum* AB161 strain (CGMCC No. 22782) was isolated from the white sediment of naturally fermented wine and identified by 16S rDNA sequencing analysis and mass spectrometry. This strain was stored in the Centre of Biological Experiments at Wenzhou Medical University.

For preparation of *Lactobacillus plantarum*-derived postbiotics, a single colony of *Lactobacillus plantarum* AB161 was cultivated in 10 mL of improved MRS liquid culture medium (Patent No. ZL 2017 1 0852478.0 in China) at 37 °C for 24–36 h. Then, the 10 mL of *Lactobacillus plantarum* suspension liquid was added to 200 mL of MRS liquid medium and cultured at 37 °C for 24–36 h. The suspension liquid containing ~10^9^ CFU/mL *Lactobacillus plantarum* was placed at 4 °C for 12–16 h, and then centrifuged at 5000 rpm at 4 °C for 10 min. The supernatant was taken and filtered through a 0.22 μm Millipore filter to obtain LP-cs (defined as LP-derived postbiotics).

### 2.2. Determination of SCFAs in LP-cs by Gas Chromatography-Mass Spectrometry (GC-MS)

The mixed standard for GC-MS analysis contained ten short-chain fatty acids (formic acid, acetic acid, propionic acid, isopropionic acid, butyric acid, isobutyric acid, valeric acid, isovaleric acid, hexanoic acid, and heptanic acid). Standard solutions were prepared after dilution using a concentration gradient. Preparation of samples: One hundred microlitres of LP-cs was pretreated by centrifugation at 13,000 rpm for 10 min at 4 °C, and then filtered through a 0.22 μm Millipore filter. The pretreated LP-cs was mixed with 300 μL of n-hexane/methanol and centrifuged at 13,000 rpm for 5 min at 4 °C. Subsequently, 100 μL of supernatant was taken and mixed with 150 μL of acetone.

GC-MS detection was performed on a GCMS QP2010 (Shimadzu, Kyoto, Japan) with an InteCap Pure-WAX column (0.25 mm × 30 m, Shimadzu, Japan). The column flow speed was 2.00 mL/min. The initial column temperature was 100 °C, and the inlet temperature was 230 °C. The injection volume was 2 μL, and the total duration time was 16 min. The mass spectrometer ion source temperature was 230 °C, the ionization mode was electron ionization (EI), the electron energy was 70 eV, the interface temperature was 250 °C, and the solvent delay time was 3 min. The detector scan rate was 714/s, the scanning area range (M/Z) was 50–400, and the data collection time was 3–7 min.

### 2.3. Electrophoresis of LP-cs by SDS-Polyacrylamide Gel Electrophoresis (SDS-PAGE)

SDS-PAGE was performed to observe the protein composition of LP-cs. Briefly, 20 μL of LP-cs was loaded in the wells of a 20% SDS-PAGE gel and electrophoresed at 70 V for 6 h on ice. The electrophoresis buffer formula was 3 g of Tris, 14.4 g of glycine, 0.1% SDS, and double distilled water was added up to 1 L. The gel was stained with Coomassie brilliant blue and imaged on a SYSTEM GelDoc XR+IMAGELAB gel imaging system (Bio-Rad, Hercules, CA, USA).

### 2.4. Preparation of LP-cs-Gel

Freeze-stored LP-cs at −80 °C were quickly placed in vacuum freeze-drying equipment for 24–36 h to prepare 10-fold concentrated LP-cs. One hundred millilitres of the 10-fold concentrated LP-cs was mixed with 200 mg of sodium alginate powder to prepare the LP-cs solution containing 2% (*w*/*v*) sodium alginate. Then, the mixed solution was slowly added to an equal amount of 2% calcium chloride solution to form calcium alginate microspheres containing 5-fold concentrated LP-cs, which were LP-cs-Gel, the calcium alginate hydrogel loaded with LP-cs, used in this study. In this process, the number of microspheres in 1 mL of solution was recorded. All solutions and materials were sterile.

### 2.5. Cell Line and Cell Culture

HL7702 cells, a human hepatocyte cell line, were cultured in RPMI-1640 containing 10% foetal bovine serum (FBS, Gibco, New York, NY, USA), 100 U/mL penicillin solution, and 0.1 mg/mL streptomycin solution at 37 °C and 5% CO_2_.

### 2.6. Evaluation of the Protective Effects of LP-cs on CCl_4_- and Alcohol-Induced Cell Injury

A certain dose of carbon tetrachloride (CCl_4_) is usually utilized in vivo to establish an animal model of acute liver injury [21]. In the current study, 10 mmol/L CCl_4_ was used to treat HL7702 cells to establish liver cell injury in vitro. Additionally, 50% alcohol was used to treat HL7702 cells to establish the cell model for acute alcoholic injury.

First, 1 × 10^5^ cells/mL/well were placed in 6-well plates. After cell adhesion, the cells were cultured on the medium containing LP-cs (dilution ratios were 1:20, 1:50, 1:100, and 1:200) and MRS medium (1:20) at 37 °C with 5% CO_2_ for 24 h, sequentially. Then, the cell medium was discarded, and the cells were washed three times with PBS. In the CCl_4_-induced cell injury model, 10 mmol/L CCl_4_ was applied to HL7702 cells for 2 h. In the alcohol-induced cell injury model, HL7702 cells were treated with 50% alcohol for 2 h. The cell survival rate assay was performed with a Cell Counting Kit-8 (Dojindo, Kumamoto, Japan).

### 2.7. Animals

Thirty male C57BL/6 mice were divided into five groups: the control group, ALI group, MRS medium-presupplemented group, LP-cs-presupplemented group, and LP-cs-Gel-presupplemented group. In the three-week pretreatment stage, all mice were fed a normal diet. The mice in the control group and ALI group drank water normally, and the mice in the MRS medium-presupplemented group and LP-cs-presupplemented group drank water containing MRS medium (1:20 dilution) and LP-cs (1:20 dilution), respectively. The mice in the LP-cs-Gel-presupplemented group were given 0.1 mL of LP-cs-Gel by gavage once every three days. The body weight of the mice was measured once a week, and the consumption of water and diet was monitored daily.

Three weeks after the above pretreatment, intragastric gavage with 50% alcohol was performed in the ALI group, MRS medium-presupplemented group, LP-cs-presupplemented group, and LP-cs-Gel-presupplemented group, at a dose of 6 g/kg B.W. The controls were treated with the same amount of 0.9% sodium chloride. Six hours later, 4% chloral hydrate was injected intraperitoneally at a dose of 0.2 mL/20 g. After anaesthesia, blood from the inner canthus was taken, and the cervical vertebra was dislocated. Ileum and liver tissues were immediately removed and embedded in OCT gel, and the remaining liver and colon contents were stored at −80 °C. The procedures of the current study were approved by the Experimental Animal Ethics Committee of Wenzhou Medical University. All experimental mice were treated in accordance with the regulations and requirements of the Experimental Animal Center of Wenzhou Medical University. This study was approved by the Ethics Committee of Experimental Animals of Wenzhou Medical University on 9 June 2020 (Project identification code: wydw2020-0777).

### 2.8. Chemical Analysis

Blood samples were incubated at 25 °C for 1 h and centrifuged at 2000× *g* at 4 °C for 10 min to obtain serum. Liver tissues (0.2 g) from each group and 1.8 mL of 0.9% normal saline were mixed and further homogenized in a homogenizer at 4 °C. The tissue homogenate was centrifuged at 2500 rpm for 10 min, and the supernatant was collected. Then, the alanine aminotransferase (ALT), aspartate aminotransferase (AST), triglyceride (TG), total cholesterol (TC), and superoxide dismutase (SOD) activities were determined according to the manufacturer instructions (Institute of Biological Engineering, Nanjing Jiancheng, China).

### 2.9. Histological Staining

First, tissue slices were prepared. Each ileum or liver tissue sample was placed on a sample tray coated with O.C.T. Compound (SAKURA, Torrance, Japan) and immediately cooled for 5–10 min to allow for the OCT glue to saturate the tissue. The tissue was continuously sliced to 5–10 μm with a frozen slicer. The slices adhered to the slides were soaked with 60% isopropyl alcohol for 2 s, and then stained with haematoxylin and eosin (H&E) and Oil red O. Photographs were captured on a Nikon DS-U3 imaging system (Nikon, Tokyo, Japan).

### 2.10. Metagenomics Analysis of Intestinal Microbiota

Fecal samples were collected, and the intestinal microflora metagenomic analysis based on 16S rDNA sequencing was performed by entrusting Shaanxi Airui Biotechnology Co., Ltd. (Xi’an, China). Briefly, genomic DNA of colon tissues was extracted, and a DNA library was constructed by PCR. Sequencing was performed on a NovaSeq6000 system (Illumina, San Diego, CA, USA). The sequence was clustered into OTUs (operational taxonomic units) with 97% consistency, and the OTU sequences were annotated by the Mothur and SILVA 138.1 databases. OTU abundance, Venn diagram, and alpha and beta diversity analyses were performed, and the structure of the microbiota in the different groups was explored. Student *t*-test, Simper, and MetaStat analyses were applied to test the significant differences in strain composition and the microbiota structure of the samples. Tax4Fun was used to perform functional prediction analysis on the microbiome.

### 2.11. Transcriptome Analysis of the Liver Tissues

Total RNA was extracted using TRIzol (Invitrogen, San Diego, CA, USA). Briefly, 1 mL of TRIzol solution was mixed with liver tissues in a 1.5 mL EP tube, and the tissues were homogenized on ice. The homogenate was transferred to a new 1.5 mL EP tube, and 0.2 mL chloroform solution was added. After 15 s of intense vortexing and 15 min of centrifugation at 12,000 rpm, the supernatant solution was carefully placed into a new 1.5 mL EP tube, and isopropyl alcohol solution was mixed at the same volume. After standing at 4 °C for 10 min and centrifugation for 10 min at 12,000 rpm, the supernatant was discarded, and 75% ethanol was added to wash the precipitate. After centrifugation, the supernatant was discarded, and the precipitate was dried for 7–30 min. Then, 20 μL of RNase-free water was used to dissolve the precipitate. The concentration and quality of RNA samples were measured on a NanoDrop 2000 (ThermoFisher, Waltham, MA, USA).

Complete mRNA library construction and sequencing analysis were performed by Shaanxi Arui Biotechnology Co., Ltd. According to the sequencing data, the transcript information and quantitative gene expression were analyzed, as well as the GO function enrichment and KEGG pathway enrichment.

### 2.12. Reverse Transcription Reaction and Quantitative PCR

Total RNA was reverse transcribed to cDNA using an HiScript^1st^ Strand cDNA Synthesis Kit (Vazyme, Nanjing, China). The thermal conditions were 42 °C for 15 min and 85 °C for 5 s. The total PCR volume was 10 μL, containing 5 μL of 2×SYBR Green Mix, 0.5 μL of each primer at 10 µM, 2 μL of H_2_O and 2 μL of cDNA template. The PCR conditions were as follows: one cycle at 95 °C for 5 min, followed by 40 cycles alternating between 95 °C for 10 s and 60 °C for 30 s. Thermal cycling was performed on a QuantStudio real-time PCR system (ThermoFisher, USA). The sequences of all primers used in this study are listed in Supplementary Table S1. The primers were synthesized by Genscript Inc. (Nanjing, China).

### 2.13. Statistical Analysis

All variables are expressed as the mean ± SEM. Comparisons were performed by Tukey’s test or one-way ANOVA. A *p*-value less than 0.05 was considered statistically significant. Statistical analyses were performed using the SPSS 26.0 statistical package and GraphPad Prime 8.0.

## 3. Results

### 3.1. SCFAs and Proteins in LP-cs

**Figure 1:**
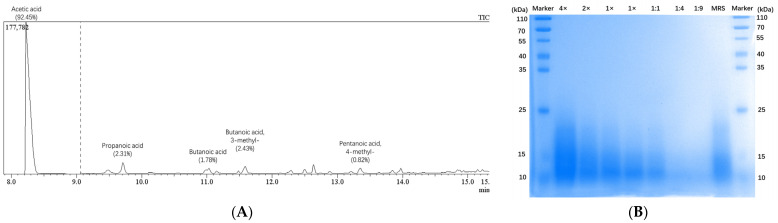
Detection of SCFAs and proteins in LP-csL: (**A**) Chromatographic result of the SCFAs in LP-cs; The main SCFAs were acetic acid; propanoic acid; butanoic acid; and butanoic acid, 3-methyl-, accounting for 92.45%, 2.31%, 1.78%, and 2.43%, respectively; the data in details were listed in Table 1; (**B**) protein bands of LP-cs in SDS-PAGE gel stained using Coomassie brilliant blue. (4× and 2×) concentrated LP-cs with 4- and 2-fold concentration; (1×) LP-cs without concentration and dilution; (1:1, 1:4, and 1:9) the diluted LP-cs with 1:1, 1:4, and 1:9 dilution rate; (MRS) MRS liquid culture medium.

SCFAs in LP-cs were detected by GC-MS. The chromatographic results showed that the main SCFAs were acetic acid/acetate (accounting for 92.45%), butyric acid/butyrate (1.78%, 2.43%), propionic acid/propionate (2.31%), and a small amount of valeric acid/valerate (0.82%, Figure 1A, Table 1). The proteins of LP-cs were mainly derived from the cell components of dead thallus or the secretions of *Lactobacillus plantarum*, or the small molecules in MRS medium, therefore, in the SDS-PAGE gel, the molecular weight of proteins in LP-cs showed a range with 10–25 kD. Moreover, the total concentration of proteins in LP-cs was lower as compared with that in MRS medium (1× vs. MRS in the gel, Figure 1B).

### 3.2. The Protective Effect of LP-cs on Cells Treated with CCl_4_ or Alcohol In Vitro

To evaluate the protective effects of LP-cs on alcohol-induced hepatocytic injury, HL7702 cells were preincubated with LP-cs, and then treated with 10 mmol/L CCl_4_ or 50% alcohol for 2 h. As a control, a group of cells were preincubated with MRS medium. 

**Figure 2:**
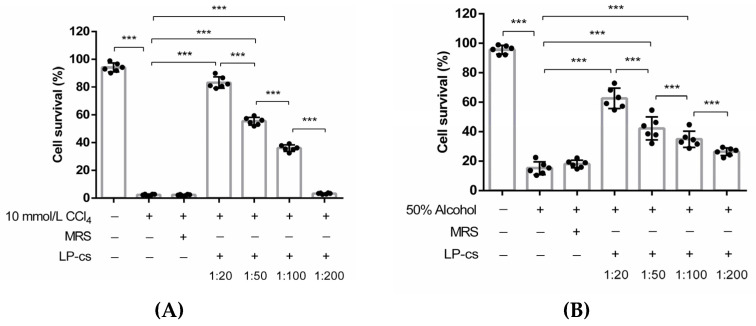
The survival rate of HL7702 cells in vitro: (**A**) The survival rates of HL7702 cells in the negative control group, 10 mmol/L CCl_4_-treated group, MRS-medium-pretreated group, and LP-cs-pretreated groups. The cell survival rates were 94.15 ± 1.32%, 2.48 ± 0.22%, 2.42 ± 0.15%, 83.25 ± 1.69%, 55.42 ± 1.13%, 36.04 ± 0.96%, and 3.35 ± 0.16%, respectively; (**B**) The survival rates of HL7702 cells in the negative control, 50% alcohol-treated group, MRS-medium-pretreated group, and LP-cs-pretreated groups. The cell survival rates were 95.62 ± 1.21%, 15.22 ± 1.77%, 18.05 ± 1.08%, 62.62 ± 2.84%, 42.23 ± 3.21%, 34.83 ± 2.25%, and 26.34 ± 1.07%, respectively. Data are presented as individual data points as well as the mean ± SEM for each group, with *n* = 6 per group. *P*-values were determined by one-way ANOVA. *** *p* < 0.001.

In the CCl_4_-induced cell injury models, the survival rates of the cells pretreated with LP-cs at 1:20, 1:50, and 1:00 dilutions were significantly increased as compared with those of the CCl_4_-treated cells and MRS-pretreated cells (*p* < 0.001, Figure 2A). Similarly, in the alcohol-induced cell injury models, the survival rates of the cells pretreated with LP-cs at 1:20, 1:50, and 1:00 dilutions were significantly increased as compared with those of the alcohol-treated cells and MRS-pretreated cells (*p* < 0.001, Figure 2B). The cell survival rates were dependent on the dilution ratio of LP-cs (Figure 2A,B). There were no differences between the CCl_4_- or alcohol-treated cells and MRS-pretreated cells. The results suggest that LP-cs alleviate the hepatocyte injury induced by 10 mmol/L CCl_4_ and 50% alcohol. MRS did not show a protective effect on the cells.

### 3.3. LP-cs Maintained the Normal Morphology of the Liver and Intestine after Acute Alcohol Intake

**Figure 3:**
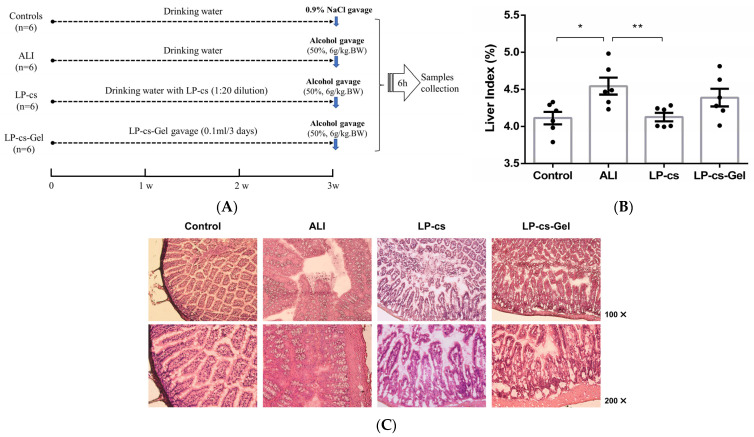
Animal experimental settings and morphology of the liver and intestine: (**A**) Animal experimental settings and schedules; (**B**) the liver index in each group of mice; the ratios were 0.041 ± 0.001, 0.045 ± 0.001, 0.041 ± 0.001, 0.044 ± 0.001 in the control, ALI, LP-cs, and LP-cs-Gel-presupplemented groups, respectively; (**C**) morphology of the proximal ileum by HE staining in each group of mice. Data are presented as individual data points as well as the mean ± SEM for each group, with *n* = 6 per group. *P*-values were determined by one-way ANOVA. * *p* < 0.05 and ** *p* < 0.01.

The animal experimental setting and schedule are presented in Figure 3A. According to the weight gain and consumption of daily diet and water of the mice during the first 3 weeks (Supplementary Figure S1, Table S2), LP-cs and LP-cs-Gel were safe for the mice. There were no abnormal conditions in the mice. 

According to the appearance, the livers in the ALI group showed significant oedema; therefore, the liver index, i.e., the ratio of liver weight to body weight, was calculated for all mice. As data showed in Figure 3B, the ratio was increased in the ALI group and decreased significantly in the LP-CS-presupplemented group (*p* < 0.05). The ratio in LP-cs-Gel-presupplemented group was higher than that in the LP-CS-presupplemented group, but there was no significant difference between the two groups. 

Considering that LP-cs contain rich SCFAs, which play an important role in maintaining normal intestinal function and the morphology and function of colonic epithelial cells, the intestinal morphology was observed by HE staining. In contrast to the controls, there was a large area of necrosis and shedding of intestinal mucosal epithelial cells, notable intestinal villi oedema, and microvascular rupture in the ALI group. However, the intestinal villi were arranged neatly, and there was mild oedemaedema of the intestinal wall in the LP-cs and LP-cs-Gel-presupplemented groups (Figure 3C). 

In addition, there were no significant differences in the liver index and intestinal morphology between the ALI group and the MRS medium-presupplemented group (data not shown).

### 3.4. LP-cs Protected Liver from Oxidative Injury

Serum ALT and AST are two key indicators of liver injury. ALT is mainly distributed in the cytoplasm of hepatocytes, and 70% of AST is distributed in the mitochondria of hepatocytes. The increase in serum ALT mainly reflects the damage of hepatocyte membrane. When the mitochondria of liver are damaged, AST will increase significantly. Therefore, if the ratio of AST/ALT increases and exceeds 1.5, it indicates serious liver damage [22].

**Figure 4:**
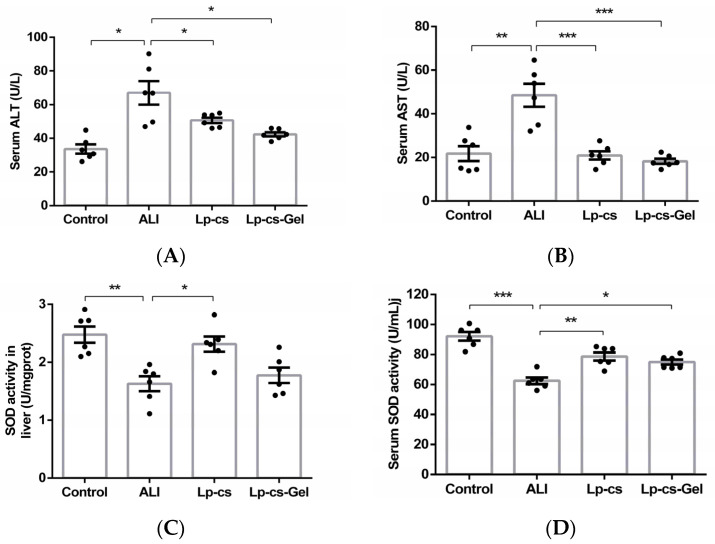
Evaluation of the liver oxidative damage in the control, ALI, LP-cs, and LP-cs-Gel-presupplemented groups: (**A**) Serum ALT levels in each group of mice, which were 33.63 ± 2.74, 67.00 ± 6.92, 50.68 ± 1.64, and 42.37 ± 1.25 U/L, respectively; (**B**) serum AST levels in each group of mice, which were 21.77 ± 3.43, 48.48 ± 5.28, 20.92 ± 1.89, and 18.26 ± 1.15 U/L, respectively; (**C**) SOD activity in the liver of the mice, which were 2.48 ± 0.14, 1.63 ± 0.13, 2.31 ± 0.13, and 1.78 ± 0.13 U/mg, respectively; (**D**) serum SOD activity in each group of mice, which were 92.07 ± 2.84, 62.47 ± 2.20, 78.68 ± 2.69, 74.96 ± 1.74, respectively. ALI, acute alcohol injury; LP-cs, *Lactobacillus plantarum* culture supernatant; LP-cs-Gel, LP-cs-loaded calcium alginate hydrogel. Data are presented as individual data points as well as the mean ± SEM for each group, with *n* = 6 per group. *p*-values were determined by one-way ANOVA. * *p* < 0.05, ** *p* < 0.01, and *** *p* < 0.001.

The levels of serum ALT and AST in the ALI group were significantly higher than those in the control group (*p* < 0.05). However, the serum AST and ALT levels in the LP-cs and LP-cs-Gel-presupplemented groups sharply decreased and restored to the normal levels (Figure 4A,B). The ratio of AST/ALT in each group was less than 1.5. To assess oxidative stress, SOD activity in the liver and serum was measured. As compared with those in the control group, the liver and serum SOD activities were significantly decreased in the ALI group (*p* < 0.01). In the LP-cs and LP-cs-Gel-presupplemented groups, SOD activities were significantly increased as compared with those in the ALI group (*p* < 0.01), and also restored to the normal levels (Figure 4C,D). There appeared to be no significant difference between LP-cs and LP-cs-Gel.

### 3.5. LP-cs Was Involved in Regulating the Expression of the Genes in the Lipid Metabolic Pathway after Acute Alchol Intake

**Figure 5:**
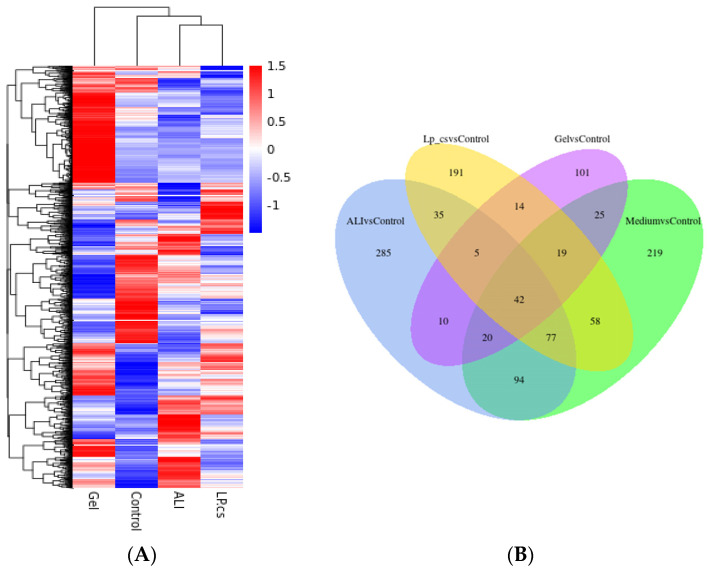

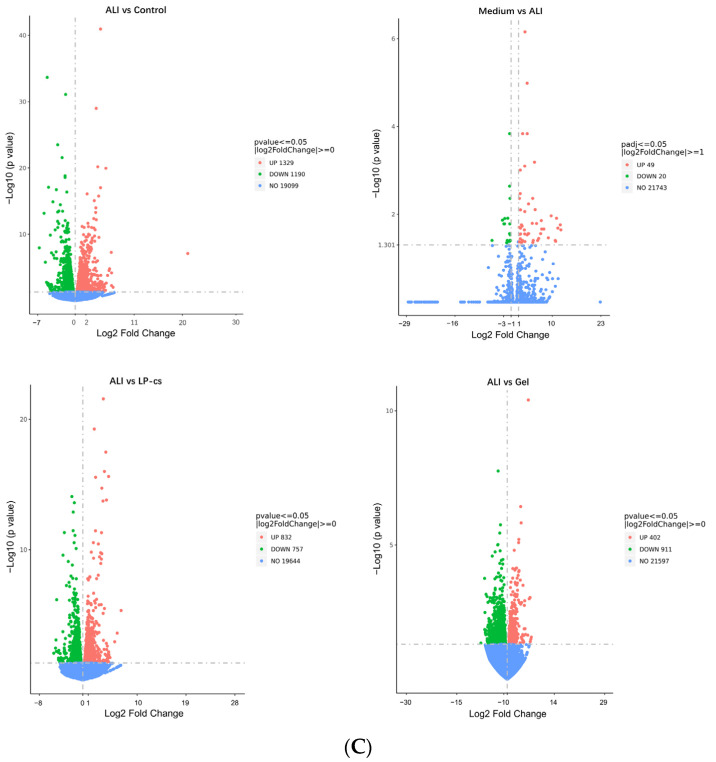
Transcriptomics analysis of liver tissues: (**A**) Heat cluster of gene expression; (**B**) Venn diagram; (**C**) volcano maps. *n* = 3 per group.

To explore the specific effects of LP-cs and the underlying molecular mechanisms, a liver transcriptomic analysis was performed. In the heat cluster map (Figure 5A), the gene expression of the ALI group and LP-cs-presupplemented group showed significant differences as compared with the control, and a very different clustering was observed in the LP-cs-Gel-presupplemented group, which indicated that there might be significant differences between LP-cs-Gel and LP-cs in the effect and molecular regulation mechanism.

The Venn map showed that the number of the overlapped genes were mostly between ALI vs. control and Medium vs. control (Figure 5B). According to the volcano plot (Figure 5C), as compared with the ALI group, the number of upregulated genes was 49, the number of downregulated genes was 20, and the number of unchanged genes was 21,743 in the MRS medium-presupplemented group. The results suggested that MRS medium had little effect on ALI mice. Therefore, this study mainly focused on the other four groups of mice.

As the volcano map showed, as compared with the control group, 1329 genes were upregulated and 1190 genes were downregulated in the ALI group. Correspondingly, as compared with the ALI group, the numbers of upregulated and downregulated genes were 832 and 757, respectively, in the LP-cs-presupplemented group; the numbers of upregulated and downregulated genes were 402 and 911 in the LP-cs-Gel-presupplemented group (Figure 5C). Moreover, the fold change in the differential genes in the LP-cs-Gel-presupplemented group was smaller than that in the LP-cs-presupplemented group (Figure 5C).

According to the KEGG enrichment (Table 2, Supplementary Figure S2), in the ALI group, the genes related to the PPAR signaling pathway, fatty acid degradation, and peroxisome cholesterol metabolism process were significantly downregulated, and the genes related to protein biosynthesis in the endoplasmic reticulum and steroid biosynthesis were significantly upregulated as compared with those in the control group. In the LP-cs-presupplemented group, the genes related to bile acid secretion, the PPAR signaling pathway, and fatty acid degradation were significantly upregulated, and the genes related to protein processing in the endoplasmic reticulum and steroid biosynthesis were significantly downregulated as compared with those the ALI group. The data of the LP-cs-Gel-presupplemented group were more significantly presented in the downregulation of steroid and steroid hormone biosynthesis-related genes as compared with those the ALI group.

According to the GO enrichment (Table 2, Supplementary Figure S3), the genes related to fatty acid metabolic processes and oxidation were significantly downregulated in the ALI group. The upregulated genes were mostly enriched in response to endoplasmic reticulum stress in the ALI group. In the LP-cs-presupplemented group, the upregulated genes were enriched in the pathways involved in small-molecule catabolic process, fatty acid metabolic process, and fatty acid oxidation; the genes related to sterol biosynthetic process, response to endoplasmic reticulum stress, and secondary alcohol biosynthetic process were significantly downregulated. In the LP-cs-Gel-presupplemented group, the upregulated genes were mostly enriched in mitochondrial respiratory chain complex assembly, and the downregulated genes were mainly enriched in sterol, secondary alcohol, and cholesterol biosynthetic processes.

Overall, the KEGG and GO enrichment results show that alcohol induced abnormal expression of the genes, mainly involved in the processes of protein biosynthesis and lipid metabolism. As compared with ALI, LP-cs presupplementation promoted the expression of genes involved in bile acid secretion, the PPAR signaling pathway (involved in lipid metabolism), and fatty acid metabolism, and downregulated the expression of steroid biosynthesis-related genes. Although the fold change in gene expression induced by LP-cs-Gel was relatively low (Figure 5C), it was significantly focused on the downregulation of genes related to steroid and cholesterol metabolism. The results also suggested that the regulation of LP in lipid metabolism should be investigated in our further research.

### 3.6. LP-cs Reduced Lipid Accumulation after Acute Alcohol Intake

**Figure 6:**
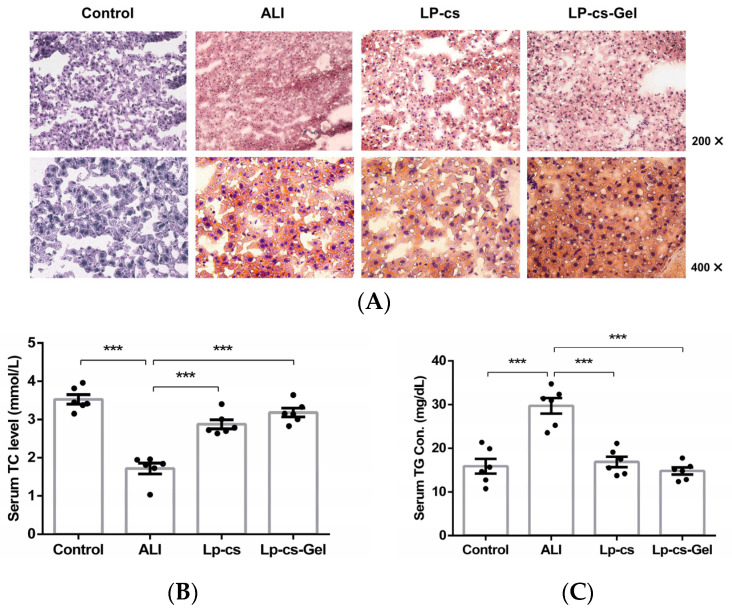
Assessment of lipid metabolism at the tissue and serum levels: (**A**) Liver tissues stained by Oil red O in each group of mice (200× and 400×); (**B**) serum TC levels in each group of mice, which were 3.53 ± 0.12, 1.72 ± 0.14, 2.88 ± 0.12, and 3.18 ± 0.11 mmol/L in the control, ALI, LP-cs, and LP-cs-Gel-presupplemented groups, respectively; (**C**) serum TG levels in the control, ALI, LP-cs, and LP-cs-Gel-presupplemented groups, which were 15.87 ± 1.67, 29.71 ± 1.78, 16.89 ± 1.19, and 14.81 ± 0.81 mg/dL, respectively. Data are presented as individual data points as well as the mean ± SEM for each group, with *n* = 6 per group. *P*-values were determined by one-way ANOVA. *** *p* < 0.001.

Oil red O staining of liver tissues showed that the number of orange-red-stained fat droplets was increased in the ALI group; they were diffuse, granular, and even fused into pieces, which indicated lipid accumulation. In the LP-cs and LP-cs-Gel-presupplemented groups, the numbers of orange-red-stained fat droplets were decreased, and the distributions of the fat droplets were sparser than that in the ALI group (Figure 6A). In addition, the level of serum TC was decreased, and the level of serum TG was increased significantly in the ALI group (*p* < 0.001), while serum TC and TG restored to normal levels in the LP-cs and LP-cs-Gel-presupplemented groups. There was no significant difference in the above results between the LP-cs and LP-cs-Gel-presupplemented groups (Figure 6B,C. These data suggested that alcohol induced the lipid accumulation in the liver, and LP-cs and LP-cs-Gel could mitigate the process and the abnormal lipid metabolism.

### 3.7. LP-cs Downregulated the Abnormal Expressed Genes Involved in Cholesterol Biosynthesis

According to the transcriptomics results, eight candidate genes with differential expression related to cholesterol metabolism between the ALI group and the LP-cs-presupplemented group were screened. These genes were *Hmgcr*, *Mvd*, *Fdps*, *Sqle*, *Lss*, *Cyp51*, *Msmo1*, and *Dhcr24*. Then, the expression levels of these genes were determined by RT-qPCR.

**Figure 7:**
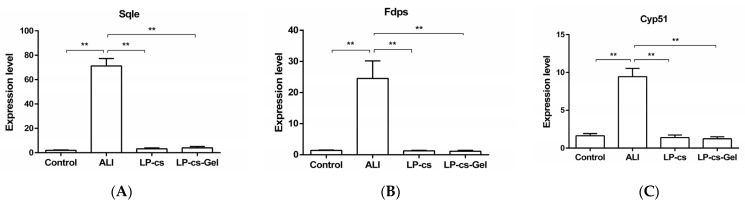
Expression levels of the genes involved in cholesterol metabolism by qPCR: (**A**–**C**) The expression levels of *Sqle*, *Msmo1,* and *Cpy51* as compared wit GAPDH expression in each group. *Sqle*, squalene epoxidase; *Msmo1*, methylsterol monooxygenase 1; *Cyp51*, cytochrome P450 family 51 subfamily. Data are presented as the mean ± SEM for each group, with *n* = 6 per group. *p*-values were determined by one-way ANOVA. ** *p* < 0.01.

The results showed that the expression levels of *Sqle*, *Msmo1*, and *Cyp51* were sharply increased in the ALI group as compared with the control group (*p* < 0.01, Figure 7). *Sqle*, encoding squalene epoxidase, which is a cholesterol synthesis rate-limiting enzyme, and *Msmo1* and *Cyp51*, encoding methylsterol monooxygenase 1 and the cytochrome P450 family 51 subfamily, respectively, are three important genes involved in cholesterol synthesis. In the LP-cs and LP-cs-Gel-presupplemented groups, the expression levels of the three genes decreased significantly as compared with those in the ALI group (*p* < 0.01, Figure 7).

### 3.8. LP-cs Restored the Diversity of the Intestinal Microbiota Community in ALI

To assess the effect of LP-cs on the structure of the intestinal microbiota, fecal samples from the mouse models were collected for metagenomic sequencing analysis. 

**Figure 8:**
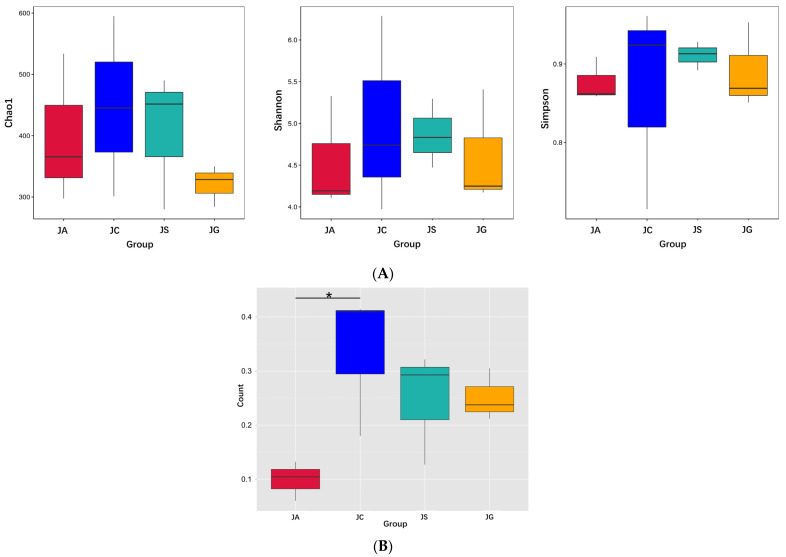
Diversity of intestinal microbiota: (**A**) Alpha diversity index of intestinal microbiota; (**B**) beta diversity index (weighted Unifrac). *n* = 3 per group. JC, control; JA, ALI; JS, LP-cs; JG, LP-cs-Gel. * *p* < 0.05.

The results of the α-diversity analysis showed that all three indexes, i.e., Chao1, Shannon, and Simpson indexes, in the ALI group were lower than those in the control group, but there were no significant differences between these two groups. The levels of three indexes in the LP-cs-presupplemented groups were similar to those in the control group. However, in the LP-cs-Gel-presupplemented groups, those indexes were more similar to those in the ALI group (Figure 8A). Chao1, Shannon, and Simpson indexes represent the total number of species, the species richness, and the species evenness of the microbiota community, respectively. The data indicated that LP-cs restored the diversity of alcohol-induced intestinal microbiota to a certain extent. However, the effects of LP-cs-Gel were not as obvious.

Additionally, the results of the β-diversity analysis showed that there was a significant difference in the weighted UniFrac distance in the intestinal microbiota between the ALI group and the control group (*p* < 0.05, Figure 8B). This explains the differences in the composition and quantity of the intestinal microbiota in the ALI group and the control group. There were no significant differences among the LP-cs, LP-cs-Gel-presupplemented groups, and the control group (Figure 8B). The results suggested that alcohol changed the community structure of intestinal microbiota in the mouse model, and P-cs and LP-cs-Gel preparations partially improved this change.

### 3.9. LP-cs Regulated the Structure of the Intestinal Microbiota

**Figure 9:**
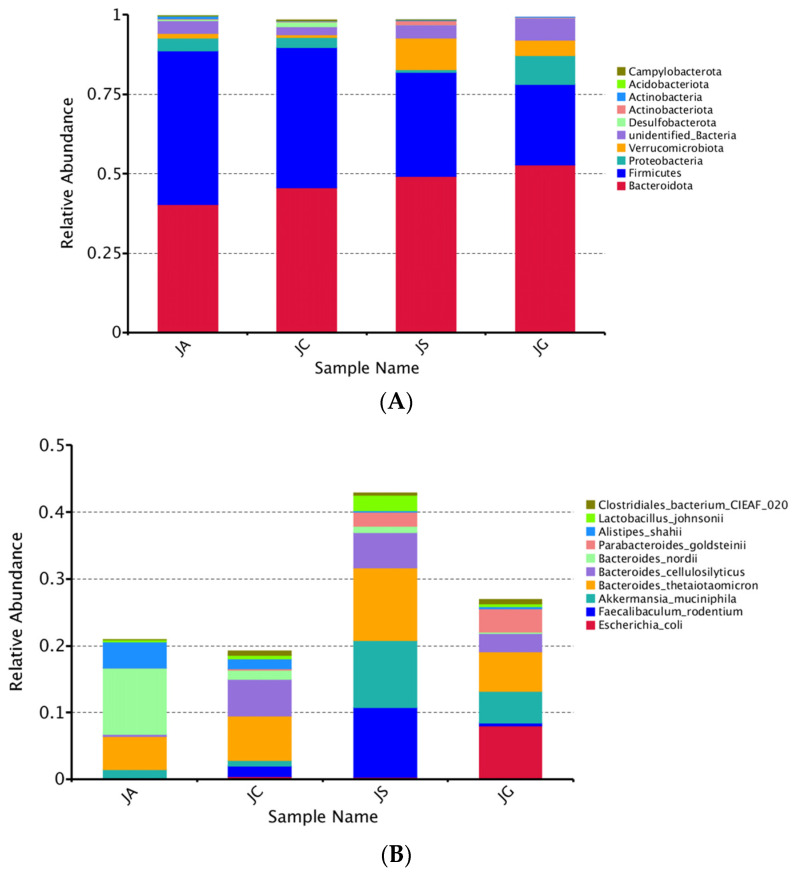

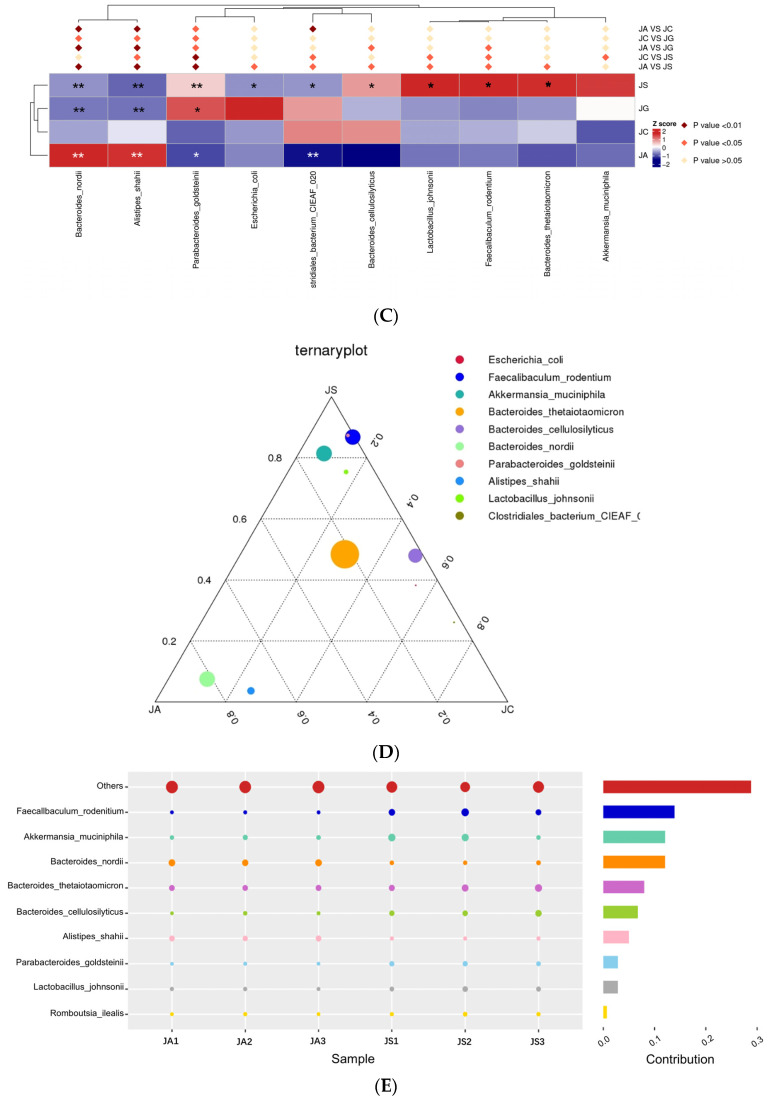
Assessment of intestinal microbiota structure based on metagenomics analysis: (**A**) Structure of intestinal microbiota at the phylum level; (**B**) the distribution of intestinal microbiota in each group at the species level (no others); (**C**) the differences between groups at the species level. Note: Black * in the graph represents the *p*-value of ALI vs. LP-cs, and white * represents the *p*-value of ALI vs. control. *P*-values between other groups are shown on the top of the clustering graph. ** *p* < 0.01 and * *p* < 0.05; (**D**) the distribution of dominant strains in the three groups (JC/JA/JS); (**E**) the contribution of strains to the difference in ALI vs. LP-cs by Simper analysis (top 10 strains). *n* = 3 per group. JC, control; JA, ALI; JS, LP-cs; JG, LP-cs-Gel. * *p* < 0.05, and ** *p* < 0.01.

The intestinal microbiota are mainly composed of five phyla, including *Firmicutes*, *Bacteroidetes*, *Actinobacteria*, *Proteobacteria,* and *Verrucomicrobia*. *Firmicutes* and *Bacteroidetes* accounted for more than 90%. In our study, the abundance of *Actinobacteriota* was significantly decreased in the ALI group as compared with that in the control group (*p* < 0.05, Table 3 and Figure 9A). The abundances of *Firmicutes* and *Actinobacteria* were significantly decreased in the LP-cs and LP-cs-Gel-presupplemented groups, and the abundances of *Bacteroidetes* in the LP-cs-Gel-presupplemented groups were significantly increased as compared with the ALI group (*p* < 0.05, Table 3, Figure 9A). 

At the species level, the total relative abundance of the top 10 strains in the LP-cs and the LP-cs-Gel-presupplemented groups were significantly increased as compared with the control group and the ALI group (Figure 9B). The abundances of *Bacteroides nordii* and *Alistipes shahii* were significantly increased, while the abundances of *Parabacteroides goldsteinii* and *Clostridiales bacterium CIEAF 020* were significantly decreased in the ALI group (*p* < 0.01, Figure 9C). As compared with the ALI group, the abundances of *Bacteroides nordii* and *Alistipes shahii* in the LP-cs-presupplemented group were significantly decreased, and the abundances of *Parabacteroides goldsteinii*, *Bacteroides cellulosilyticus*, *Lactobacillus johnsonii*, *Faecallbaculum rodentium*, and *Bacteroides thetaiotaomicron* were significantly increased (*p* < 0.05, Figure 9C). 

According to the results of the ternary plot analysis at species level, the predominant strains in the LP-cs-presupplemented group were *Faecallbaculum rodenitium*, *Akkermansia muciniphila*, and *Parabacteroides goldsteinii* (with lower abundance); that in the ALI group was *Bacteroides nordii* (Figure 9D). Similarity the percentage analysis showed that the top two strains with contributions to the difference between the ALI and LP-cs-presupplemented groups were *Faecallbaculum rodentium* and *Akkermansia muciniphila* (Figure 9E).

The above results showed that, as compared with ALI, LP-cs regulated the composition of intestinal microflora at the gate and species levels after acute alcohol intake. The regulation of LP-cs-Gel on intestinal microbiota was not significant.

### 3.10. Functional Prediction of Intestinal Microbiota Based on Metagenomic Sequencing

**Figure 10:**
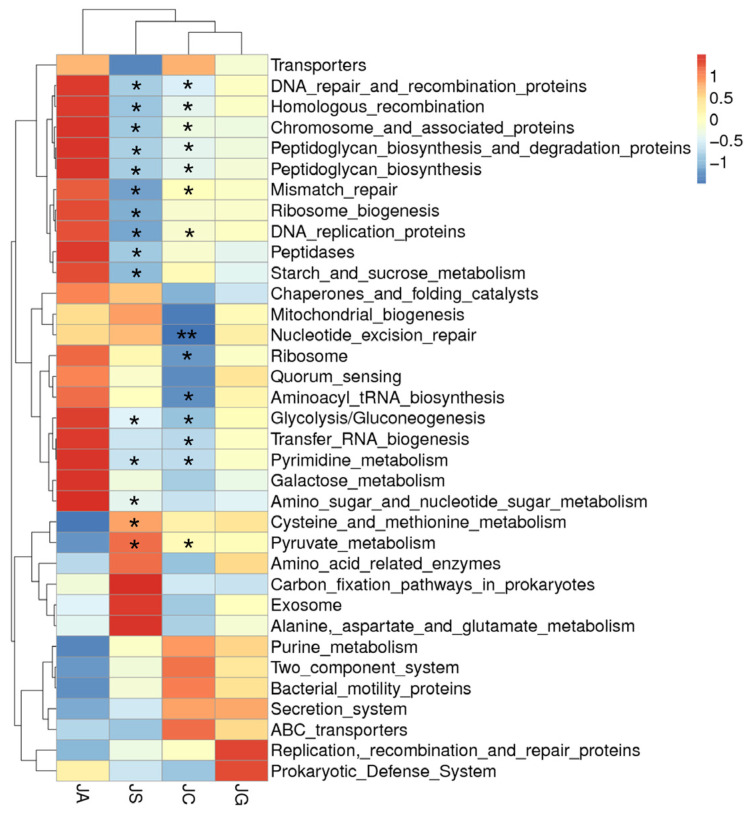
Cluster heatmap of the function prediction of strains with varying abundances. Black * marked in the graph represents the *p*-value of LP-cs vs. ALI and control vs. ALI. *n* = 3 per group. JC, control; JA, ALI; JS, LP-cs; JG, LP-cs-Gel. * *p* < 0.05, and ** *p* < 0.01.

Functional prediction of the intestinal microbiota through Tax4Fun showed that the strains with significantly increased abundance in the ALI group mainly participate in genetic information processing (including DNA recombination and repair and tRNA biosynthesis) and metabolism (including glycolysis, gluconeogenesis, and pyrimidine metabolism) (*p* < 0.05). The strains participating in these processes were significantly decreased in the LP-cs-presupplemented group as compared with those in the ALI group (*p* < 0.05, Figure 10). However, there was no significant difference in the abundance of microbiota between the LP-cs-Gel-presupplemented group and the ALI group.

## 4. Discussion

Currently, lactic acid bacteria are mainly used as probiotics, among which *Lactobacillus plantarum* is a common bacterium that exists in dairy products, fruits, and vegetables. Recent studies have found that *Lactobacillus plantarum* was beneficial to preventing and improving liver alcoholic steatosis and restoring and improving intestinal balance, thereby, reducing serum endotoxin levels [7,8,13,15,16,17,18]. However, the potential mechanism by which LAB, including *Lactobacillus plantarum*, prevents ALI has not yet been fully elucidated.

Postbiotics are derived from inanimate microorganisms and/or their components. They could be mixtures that contain proteins, peptides, SCFAs, organic acids, or vitamins. Therefore, LAB-derived postbiotics might be the potential factor by which LAB play a role in ALI treatment. Postbiotics not only overcome the selective pressure in the colonization of probiotics in the intestinal tract, but also avoid excessive immune stimulation of biological macromolecules derived from probiotics. Additionally, due to their advantages in storage stability and components with known chemical structures, the role of postbiotics in the prevention and treatment of diseases has gradually attracted attention. Postbiotics have been found to have immune regulation, anti-inflammation, and anti-adhesion abilities [23,24,25,26]. Therefore, they have been used to alleviate obesity, hypertension, coronary artery disease, and cancer [27]. Moreover, in addition to the role of LGGs in ALI treatment discussed in previous studies, a recent study found that ultrasound-treated *Lactobacillus plantarum* suspension was more effective than *Lactobacillus plantarum* thallus suspension in antioxidant activity and the prevention of alcohol-induced liver injury in zebrafish larvae [18]. In our previous study, we isolated *Lactobacillus plantarum* AB161 strain from the sediment of naturally fermented wine, and found this strain was alcohol resistant. Therefore, in this study, the role of *Lactobacillus plantarum* AB161-derived postbiotics in acute ALI was investigated. In addition, LP-cs-Gel was prepared and applied to ALI mice to evaluate the feasibility of this preparation.

First, SCFAs in LP-cs were detected by GC-MS. SCFAs in LP-cs were rich and dominated by acetic acid (92.45%), followed by butyric acid, and propionic acid (Figure 1A, Table 1). Physiologically, SCFAs are beneficial carbohydrates produced by intestinal microbiota [28]. Acetic acid, propionic acid, and butyric acid are three main components of SCFAs. Acetic acid accounts for the highest proportion of SCFAs. Acetic acid plays a role in regulating intestinal pH, maintaining the stability of the intestinal microenvironment, nourishing beneficial microorganisms, and preventing the invasion of opportunistic pathogens. In addition, acetic acid is beneficial to butyric acid-producing bacteria in the intestine and the diversity of beneficial flora. Firmicutes use acetic acid to produce butyric acid. Butyric acid is the main energy source of colon cells (>90%). Butyric acid maintains the integrity of the intestinal wall and prevents pathogenic bacteria, toxins, and other harmful substances from entering the circulation [29]. The data indicated that LP-cs might be safe and friendly for intestine. 

The assessment of cell survival clarified the safety in the application of LP-cs. According to our in vitro data, LP-cs sharply promoted cell survival even in the presence of high concentrations of alcohol and CCl_4_. (Figure 2). Different from LP-cs, MRS medium did not show the protective effect (Figure 2). In the subsequent preclinical study, during the stage of presupplementation with LP-cs or LP-cs-Gel for 3 weeks, each group of mice presented a healthy state with a steady intake of water and diet, weight gain, and a normal ratio of liver and body weight (Supplementary Figure S1 and Table S2), which also indicated that LP-cs were safe for use in mice. 

Alcohol induces excessive production of ROS and inhibits fatty acid oxidation, leading to liver oxidative injury and steatosis [11]. In this study, the presupplementation of LP-cs could maintain the normal morphology of the liver and intestine after acute alcohol intake (Figure 3). Acute alcohol intake caused liver injury, but not severe liver damage, meanwhile, LP-cs and LP-cs-Gel could restore the levels of serum AST and ALT, as well as SOD activities in the liver and serum after acute alcohol intake (Figure 4). These findings were consistent with the protective effect of postbiotics, LGGs and *Lactobacillus plantarum thallus* [9,18], and other strains of *Lactobacillus plantarum* on hepatocytes and enterocytes in ALI reported previously [8,13]. 

In further investigation, it was found that LP-cs upregulated the expression of genes involved in the PPAR signaling pathway and the fatty acid metabolic process, and downregulated the expression of genes involved in steroid/cholesterol biosynthesis in ALI (Table 2). Additionally, LP-cs reduced lipid accumulation in the liver, as well as restored the levels of serum TC and TG after acute alcohol intake (Figure 6). Additionally, the expression levels of the differentially expressed genes *Sqle*, *Msmo1,* and *Cyp51*, involved in cholesterol synthesis, coincided with the differences in transcriptomic analysis (Table 2, Figure 5 and Figure 7). The above findings indicated that both of LP-cs and LP-cs-Gel regulated the expression of the genes involved in lipid metabolic processes, and ameliorated the abnormalities in alcohol-induced lipid metabolism. Moreover, the effects of LP-cs were better than those of LP-cs-Gel. 

Alcohol-induced fat accumulation is mainly due to increased lipid production and decreased mitochondrial β-oxidation of fatty acids [30]. Alcohol intake reduces the phosphorylation of AMP-activated protein kinase (AMPK) and increases the activity of acetyl-CoA carboxylase, thereby, increasing fat accumulation in the liver [31]. Previous studies on LGGs have shown that LGGs increased the phosphorylation of AMPK, promoted β-oxidation of fatty acids, downregulated the expression of sterol regulatory element binding protein-1 (SREBP-1), and upregulated the expression of peroxisome proliferator activated receptor-α (PPAR-α). PPAR-α is a key transcriptional regulator of many genes in mitochondrial oxidation and plays a key role in regulating the enzymes responsible for the synthesis of cholesterol, fatty acids, and triglycerides in the liver [32]. Therefore, LGGs alleviate alcohol-induced lipid accumulation [12]. A study on the effect of *Lactobacillus paracasei*-derived postbiotics on rats with a high-fat diet (HFD) showed that the postbiotics could correct disorders in lipid metabolism and reduced the level of serum lipid in HFD-fed rats. The effect of *Lactobacillus paracasei*-derived postbiotics is better than that of atorvastatin (ATOR), a lipid-regulating drug [33]. In a recent study, it was found that *Akkermansia muciniphila* and its extracellular vehicles (EVs) improved obesity by regulating lipid metabolism, and reduced body weight gain and fat accumulation in HFD-fed mice [34]. Our current data also supported the positive role of LP-cs in regulating liver lipid metabolism and preventing liver lipid accumulation in ALI, but the potential molecular mechanism requires more investigations in the future.

The changes in the structure of the intestinal microbiota caused by excessive alcohol have been confirmed by a large number of studies. As early as 1984, a study by Bode et al. first described the changes in intestinal microbiota in ALD patients. Gram-negative anaerobic and aerobic bacteria were found to be significantly increased in ALD patients as compared with controls [35]. Since then, several studies have demonstrated this altered characteristic, i.e., that alcohol can lead to the excessive growth of intestinal Gram-negative bacteria [36]. They have also found that alcohol reduced the number of intestinal *Firmicutes* and *Bacteroides*, thereby, changing the ratio of *Firmicutes* and *Bacteroidetes*, which was used as a diagnostic parameter for ALD [37]. In addition, alcohol induced an increase in the number of *Actinobacteria* and *Proteobacteria* [38], and a decrease in *Lactobacillus* [39]. Moreover, alcohol led to the proliferation of *Corynebacterium* and *Alcaligenes*, which increased the intestinal pH value, and finally, led to excessive growth of intestinal pathogenic bacteria [38]. Therefore, the influence of alcohol on the structure of intestinal microbiota was explored in our current study.

Presupplementation of LP-cs improved the community structure of the intestinal microbiota, and sharply increased the total abundance of microbiota at the species level on alcoholic stress (Figure 8 and Figure 9), which might be a timely protective mechanism under alcoholic stress. Notably, the dominant strains with higher contributions to the difference between the ALI and LP-cs-presupplemented group were *Faecallbaculum rodenitium* and *Akkermansia muciniphila* (Figure 9D,E). *Akkermansia muciniphila* (*A. muciniphila*) is a strictly anaerobic Gram-negative bacterium that accounts for 1%–4% of the human intestinal flora [40]. Physiologically, *A. muciniphila* adheres to the mucus layer of the intestine, utilizes the glycan oligosaccharides as nutrients [41], and produces degradation metabolites of mucin, such as SCFAs. SCFAs have been proved to function as histone deacetylase inhibitors and regulate host lipid metabolism and the expression of immune response-related genes through epigenetic modifications [42]. Therefore, a decrease in the abundance of *A. muciniphila* leads to the thinning of the mucus layer and the impairment of intestinal barriers [43]. Studies have also proven that *A. muciniphila* significantly reduced immune-mediated liver injury [44,45] and also reversed the liver damage and the dysfunction of intestinal microbiota induced by alcohol [41]. In a previous study, the potential beneficial effects of *A. muciniphila* were investigated for the preparation of postbiotics [46].

In this study, the increased abundance of *A. muciniphila* could be associated with presupplementation of LP-cs. SCFAs in LP-cs could provide an abundant nutrient source for intestinal epithelial cells, and potentially promote intestinal epithelial cells to secrete sufficient mucus. As a result, it would maintain the function of the intestinal barrier. Additionally, *A. muciniphila* utilized mucus to grow and produce SCFAs. Moreover, the functional prediction of the intestinal microbiota showed that the microorganisms involved in the stress response, such as DNA repair, tRNA biosynthesis, and some essential metabolic processes, were not significantly increased in LP-cs-presupplemented mice after acute alcohol intake (Figure 10). These results indicated that LP-cs might be beneficial to the growth of *A. muciniphila*, more importantly, the symbiosis of intestinal microorganisms for protecting the intestine after acute alcohol intake.

In the current study, LP-cs-Gel was prepared to explore a new manner of administration, but there were still some limitations. Alginate is a natural biopolymer that is typically extracted from brown seaweeds, brown algae, and bacteria. Due to its excellent biocompatibility, permeability, cell viability, and stability, alginate has been applied in the pharmaceutical, food, and biomedical fields [47]. Calcium alginate hydrogels have been widely used in medicine as sealants, swelling agents, and promoters for wound healing or angiogenesis [48,49,50]. Oral delivery of LP-cs-Gel is safe for the mice, moreover, it seemed to be similar to LP-cs in the antioxidative capacity and protective effects. However, there was little difference between the LP-cs-Gel preparation and LP-cs in the regulation of lipid metabolism and the structure of intestinal microbiota. The fold change of gene expression, the increased abundance of the intestinal microbiota, and the response to alcoholic stress in LP-cs-presupplemented group was not as significant as those of the LP-cs-presupplemented group (Figure 5, Figure 9 and Figure 10). The possible reason for these differences might be related to the structural characteristics of PL-cs-Gel. It was possible that the contact between the calcium alginate hydrogel and the intestinal mucosa was insufficient. Alternatively, whether LP-cs was released from the calcium alginate particles depended on the intestinal pH and the complex microenvironment in the intestinal lumen. Therefore, the preparation approaches of LP-cs-Gel require more optimization.

## 5. Conclusions

*Lactobacillus plantarum*-derived postbiotic, i.e., LP-cs, is from the supernatant of *Lactobacillus plantarum* culture medium and contains abundant SCFAs, proteins, and polypeptides, as well as other potential cellular components or organic metabolites.

In our current study, LP-cs showed effectiveness in the maintenance of liver and intestinal histomorphology, antioxidation, regulation of lipid metabolism, and intestinal microbial homeostasis on alcoholic stress. Due to its abundant SCAFs, LP-cs potentially provide nutrients and promote the secretion of mucus in intestinal epithelial cells to maintain the function of the intestinal barrier. Moreover, the increased secretion of mucus is conducive to the production of more SCFAs by *A. muciniphila*. It contributes to the diversity of beneficial flora. Overall, LP-cs is beneficial to the symbiosis of intestinal microorganisms, which is important for maintaining the function of the intestine and the liver. Although the effect of LP-cs-Gel is not as obvious as that of LP-cs according to the current data, LP-cs-Gel still has great application potential, mainly due to its easy quantification and management.

## Figures and Tables

**Table 1:** SCFAs of LP-cs by GC-MS.

Components	Retention Time (min)	Mass Charge Ratio (m/z)	Peak Area (uS*min)	Peak Height (mAu*s)	Concentration (nM)	Percentage (Area, %)
Acetic acid	8.222	60	776,319	172,651	3410.682	92.45%
Propanoic acid	9.715	74	19,427	5762	105.640	2.31%
Propanoic acid,2-methyl-	10.140	73	1801	795	8.349	0.21%
Butanoic acid	11.031	60	14,959	4054	10.817	1.78%
Butanoic acid,3-methyl-	11.588	60	20,376	6148	20.486	2.43%
Pentanoic acid,4-methyl-	13.349	57	6879	2037	11.850	0.82%

**Table 2:** Up- or downregulation in KEGG and GO enrichment (top 5).

Groups		KEGG Enrichment			GO Enrichment	
		Upregulation	Downregulation		Upregulation	Downregulation
ALI vs. control	1	Protein processing in endoplasmic reticulum	PPAR signaling pathway	1	Response to endoplasmic reticulum stress	Fatty acid metabolic process
	2	Protein export	Fatty acid degradation	2	tRNA aminoacylation	Organic acid catabolic process
	3	Aminoacyl-tRNA biosynthesis	Peroxisome	3	Amino acid activation	Carboxylic acid catabolic process
	4	Terpenoid backbone biosynthesis	Fatty acid metabolism	4	tRNA aminoacylation for protein translation	Fatty acid oxidation
	5	Steroid biosynthesis	Cholesterol metabolism	5	ncRNA metabolic process	Lipid oxidation
LP-cs vs. ALI	1	Bile secretion	Protein processing in endoplasmic reticulum	1	Small molecule catabolic process	Sterol biosynthetic process
	2	PPAR signaling pathway	Steroid biosynthesis	2	Organic acid catabolic process	Response to endoplasmic reticulum stress
	3	Carbon metabolism	Terpenoid backbone biosynthesis	3	Carboxylic acid catabolic process	Secondary alcohol biosynthetic process
	4	Fatty acid degradation	Protein export	4	Fatty acid metabolic process	Cholesterol biosynthetic process
	5	Propanoate metabolism	Aminoacyl-tRNA biosynthesis	5	Fatty acid oxidation	Sterol metabolic process
LP-cs-Gel vs. ALI	1	Parkinson disease	Terpenoid backbone biosynthesis	1	Mitochondrial respiratory chain complex assembly	Sterol biosynthetic process
	2	Thermogenesis	Steroid biosynthesis	2	NADH dehydrogenase complex assembly	Secondary alcohol biosynthetic process
	3	Alzhemer disease	Steroid hormone biosynthesis	3	Mitochondrial respiratory chain complex I assembly	Cholesterol biosynthetic process
	4	Oxidative phosphorylation	Hepatitis C	4	Electron transport chain	Sterol metabolic process
	5	Hontington disease	Cell cycle	5	Cellular respiration	Steroid biosynthetic process

ALI, acute alcohol injury; LP-cs, *Lactobacillus plantarum* culture supernatant; LP-cs-Gel, LP-cs-loaded calcium alginate hydrogel

**Table 3:** Differences in composition at phylum level.

Group 1 vs. Group 2		Group 1		Group 2		*p* Value
	Phylum	Mean	Standard Error	Mean	Standard Error	
ALI vs. Control	Actinobacteriota	0.00235609	0.000301323	0.0036587	0.000496433	0.042157895
ALI vs. LP-cs	Firmicutes	0.4846569	0.027519117	0.3267776	0.043929576	0.020631579
	Actinobacteriota	0.0023561	0.000301323	0.0142291	0.000147088	0
	Actinobacteria	0.0076733	0.002852918	0.0012528	4.66765 × 10^−5^	0.030789474
ALI vs. LP-cs-Gel	Bacteroidota	0.4024714	0.003654129	0.5271662	0.020035264	0
	Firmicutes	0.4846569	0.027519117	0.2552051	0.081547202	0.006117647
	Actinobacteria	0.0076733	0.002852918	0.0008755	4.93 × 10^−5^	0.012235294

ALI, acute alcohol injury; LP-cs, *Lactobacillus plantarum* culture supernatant; LP-cs-Gel, LP-cs-loaded calcium alginate hydrogel

## Data Availability

The dataset currently is not set up, but all data in the current study is available by contacting the authors.

## Supplementary Materials

The following supporting information can be downloaded at: https://www.mdpi.com/article/10.3390/nu15040845/s1, Figure S1: The weight gain during the 3 weeks in each group of mice; Figure S2: KEGG enrichments of the differentially expressed genes; Figure S3: GO enrichments of the differentially expressed genes; Table S1: The sequence of primers for amplifying the target genes; Table S2: Mean consumption of daily water and dietary for 3 weeks in each group of mice.
