# Supplementary material for: Lactobacillus plantarum-Derived Postbiotics Ameliorate Acute Alcohol-Induced Liver Injury by Protecting Cells from Oxidative Damage, Improving Lipid Metabolism, and Regulating Intestinal Microbiota
Source: Nutrients. 2023 Feb 7;15(4):845. doi: 10.3390/nu15040845 (PMC9965849; doi:10.3390/nu15040845)
Supplement: Supplementary file 1 [file nutrients-15-00845-s001.zip › nutrients-2107819-supplementary.pdf]

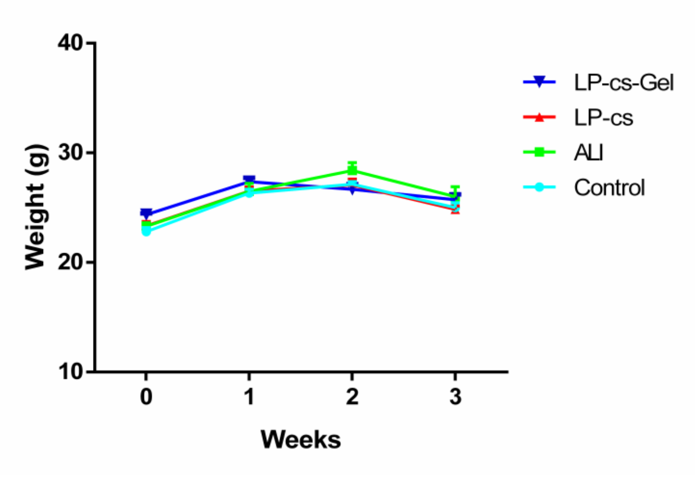

Figure S1. The weight gain during the 3 weeks in each group of mice.

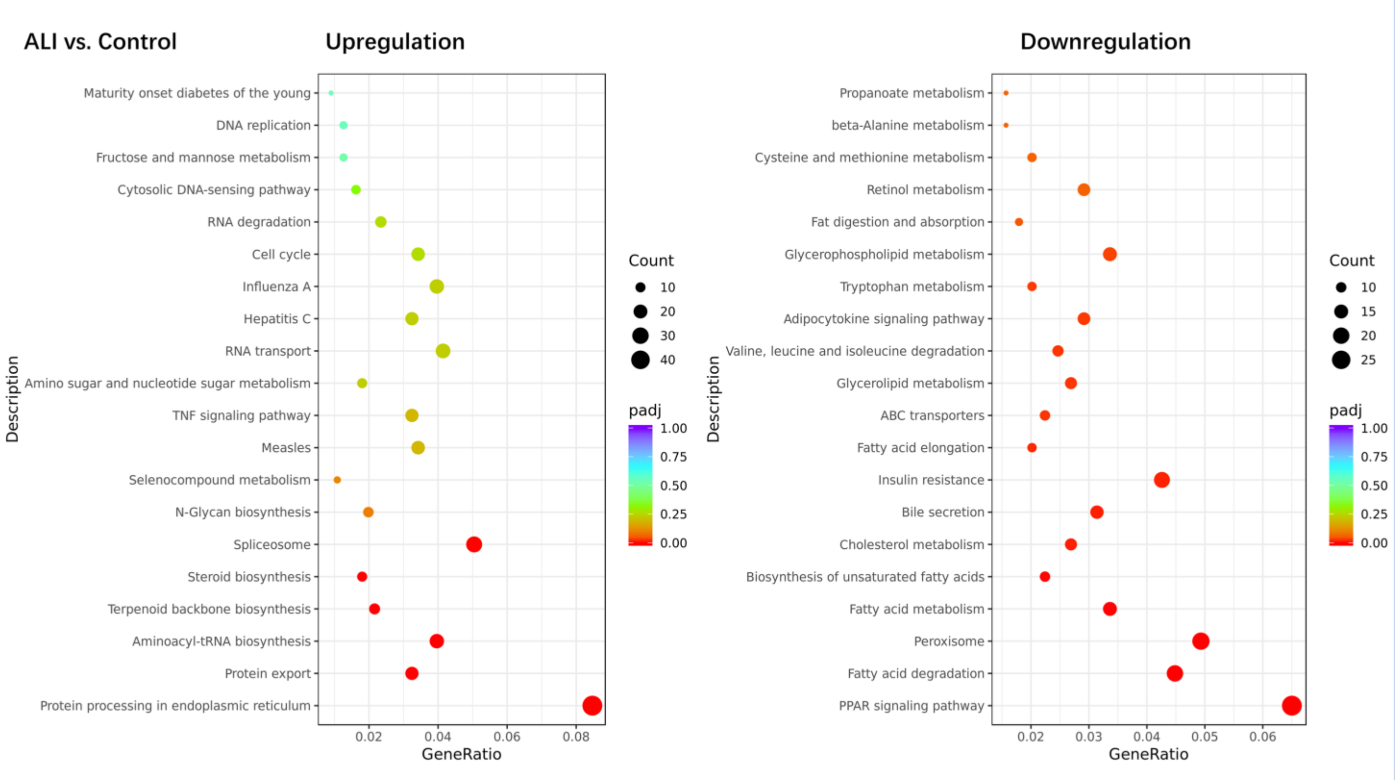

(A)

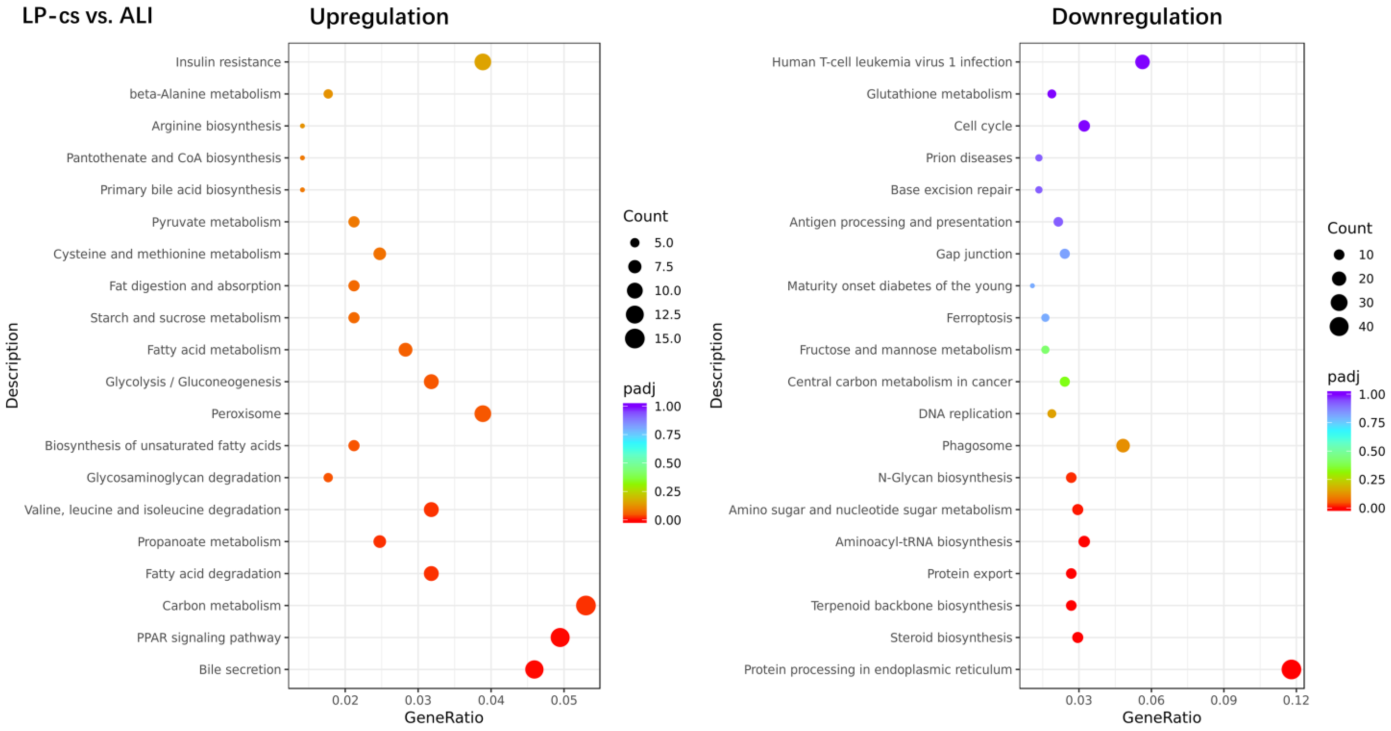

(B)

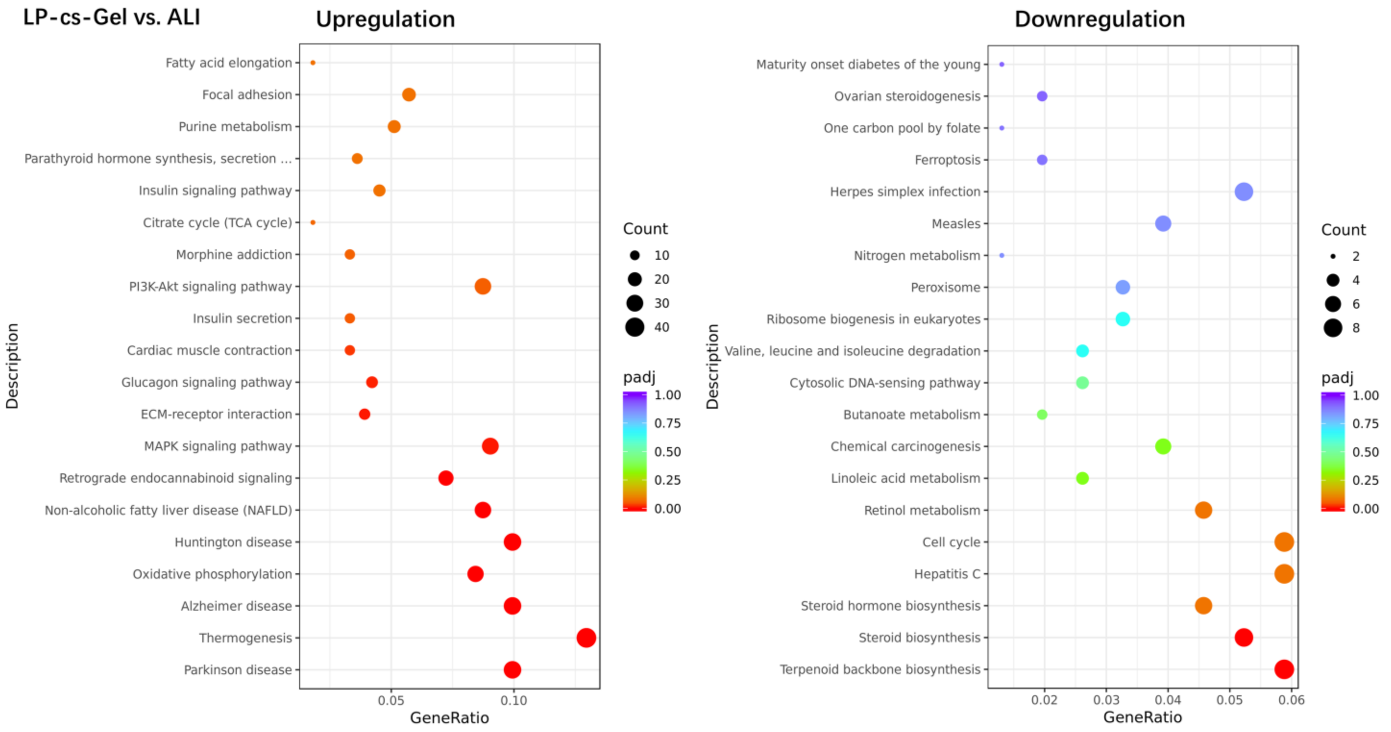

(C)

Figure S2. KEGG enrichments of the differentially expressed genes. A–C: KEGG enrichment results of (A): ALI vs. the control; (B) ALI vs. LP-cs-Gel; (C): ALI vs. LP-cs-Gel.

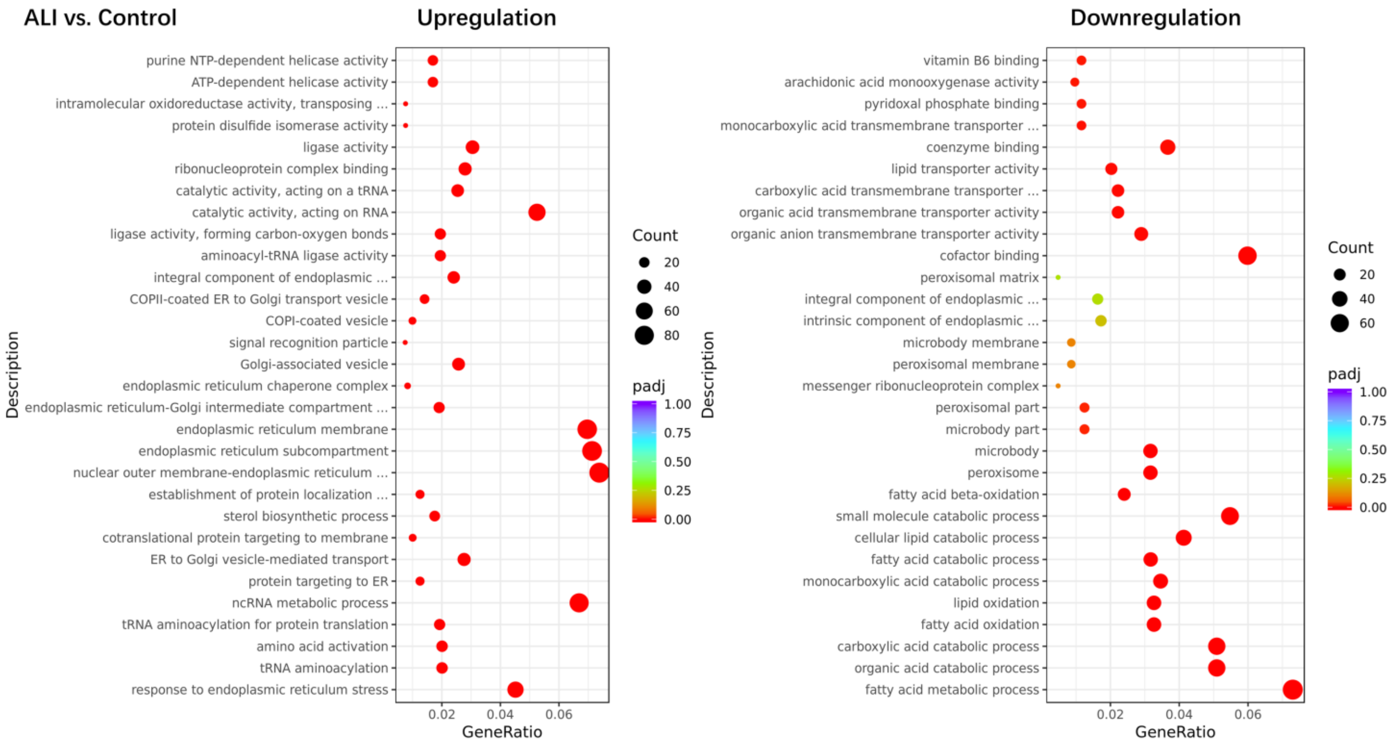

ALI vs. Control
Upregulation
purine NTP-dependent helicase activity
ATP-dependent helicase activity
intramolecular oxidoreductase activity, transposing ...
protein disulfide isomerase activity
ligase activity
ribonucleoprotein complex binding
catalytic activity, acting on a tRNA
catalytic activity, acting on RNA
ligase activity, forming carbon-oxygen bonds
aminoacyl-tRNA ligase activity
integral component of endoplasmic ...
COPII-coated ER to Golgi transport vesicle
COPI-coated vesicle
signal recognition particle
Golgi-associated vesicle
endoplasmic reticulum chaperone complex
endoplasmic reticulum-Golgi intermediate compartment ...
endoplasmic reticulum membrane
endoplasmic reticulum subcompartment
nuclear outer membrane-endoplasmic reticulum ...
establishment of protein localization ...
sterol biosynthetic process
cotranslational protein targeting to membrane
ER to Golgi vesicle-mediated transport
protein targeting to ER
ncRNA metabolic process
tRNA aminoacylation for protein translation
amino acid activation
tRNA aminoacylation
response to endoplasmic reticulum stress
Description
0.02
0.04
0.06
GeneRatio
Count
20
40
60
80
padj
1.00
0.75
0.50
0.25
0.00
Downregulation
vitamin B6 binding
arachidonic acid monooxygenase activity
pyridoxal phosphate binding
monocarboxylic acid transmembrane transporter ...
coenzyme binding
lipid transporter activity
carboxylic acid transmembrane transporter ...
organic acid transmembrane transporter activity
organic anion transmembrane transporter activity
cofactor binding
peroxisomal matrix
integral component of endoplasmic ...
intrinsic component of endoplasmic ...
microbody membrane
peroxisomal membrane
messenger ribonucleoprotein complex
peroxisomal part
microbody part
microbody
peroxisome
fatty acid beta-oxidation
small molecule catabolic process
cellular lipid catabolic process
fatty acid catabolic process
monocarboxylic acid catabolic process
lipid oxidation
fatty acid oxidation
carboxylic acid catabolic process
organic acid catabolic process
fatty acid metabolic process
Description
0.02
0.04
0.06
GeneRatio
Count
20
40
60
padj
1.00
0.75
0.50
0.25
0.00

(A)

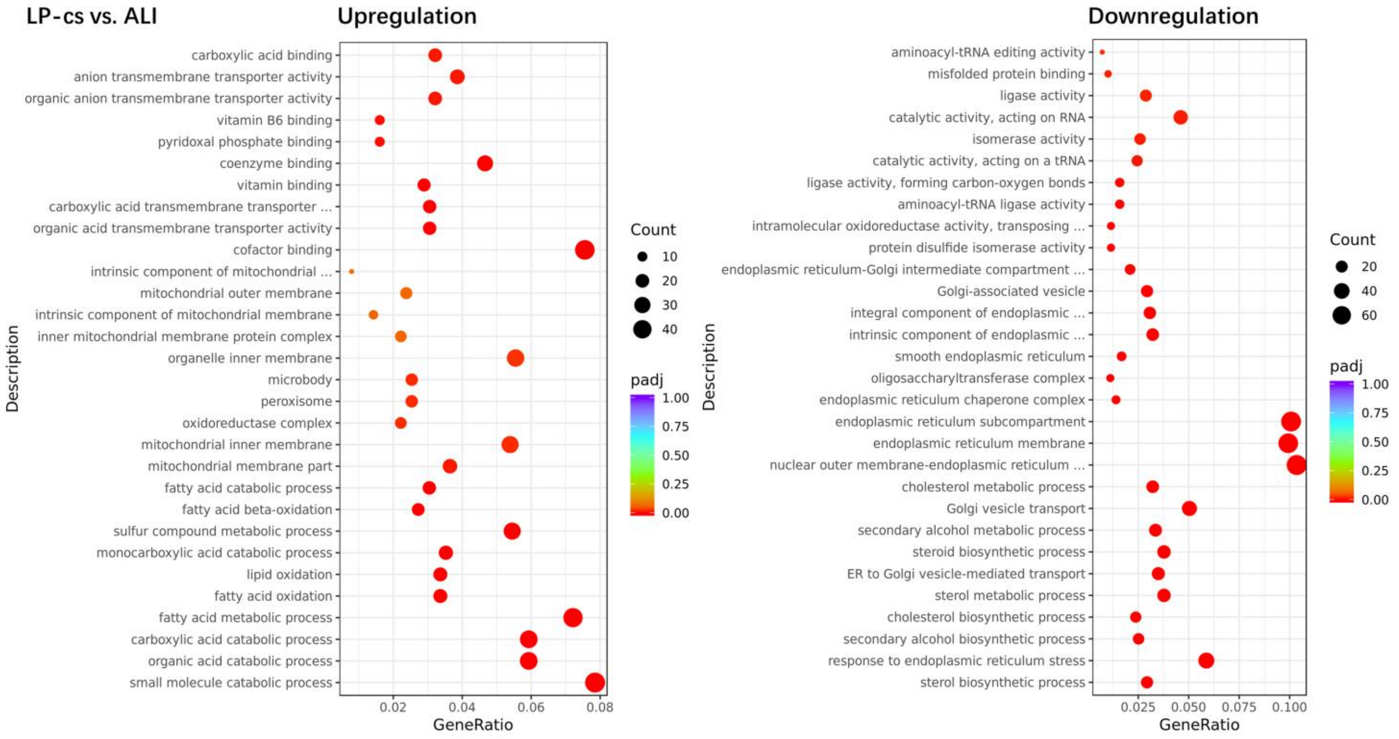

LP-cs vs. ALI
Upregulation
carboxylic acid binding
anion transmembrane transporter activity
organic anion transmembrane transporter activity
vitamin B6 binding
pyridoxal phosphate binding
coenzyme binding
vitamin binding
carboxylic acid transmembrane transporter ...
organic acid transmembrane transporter activity
cofactor binding
intrinsic component of mitochondrial ...
mitochondrial outer membrane
intrinsic component of mitochondrial membrane
inner mitochondrial membrane protein complex
organelle inner membrane
microbody
peroxisome
oxidoreductase complex
mitochondrial inner membrane
mitochondrial membrane part
fatty acid catabolic process
fatty acid beta-oxidation
sulfur compound metabolic process
monocarboxylic acid catabolic process
lipid oxidation
fatty acid oxidation
fatty acid metabolic process
carboxylic acid catabolic process
organic acid catabolic process
small molecule catabolic process
Description
0.02
0.04
0.06
0.08
GeneRatio
Count
10
20
30
40
padj
1.00
0.75
0.50
0.25
0.00
Downregulation
aminoacyl-tRNA editing activity
misfolded protein binding
ligase activity
catalytic activity, acting on RNA
isomerase activity
catalytic activity, acting on a tRNA
ligase activity, forming carbon-oxygen bonds
aminoacyl-tRNA ligase activity
intramolecular oxidoreductase activity, transposing ...
protein disulfide isomerase activity
endoplasmic reticulum-Golgi intermediate compartment ...
Golgi-associated vesicle
integral component of endoplasmic ...
intrinsic component of endoplasmic ...
smooth endoplasmic reticulum
oligosaccharyltransferase complex
endoplasmic reticulum chaperone complex
endoplasmic reticulum subcompartment
endoplasmic reticulum membrane
nuclear outer membrane-endoplasmic reticulum ...
cholesterol metabolic process
Golgi vesicle transport
secondary alcohol metabolic process
steroid biosynthetic process
ER to Golgi vesicle-mediated transport
sterol metabolic process
cholesterol biosynthetic process
secondary alcohol biosynthetic process
response to endoplasmic reticulum stress
sterol biosynthetic process
Description
0.025
0.050
0.075
0.100
GeneRatio
Count
20
40
60
padj
1.00
0.75
0.50
0.25
0.00

(B)

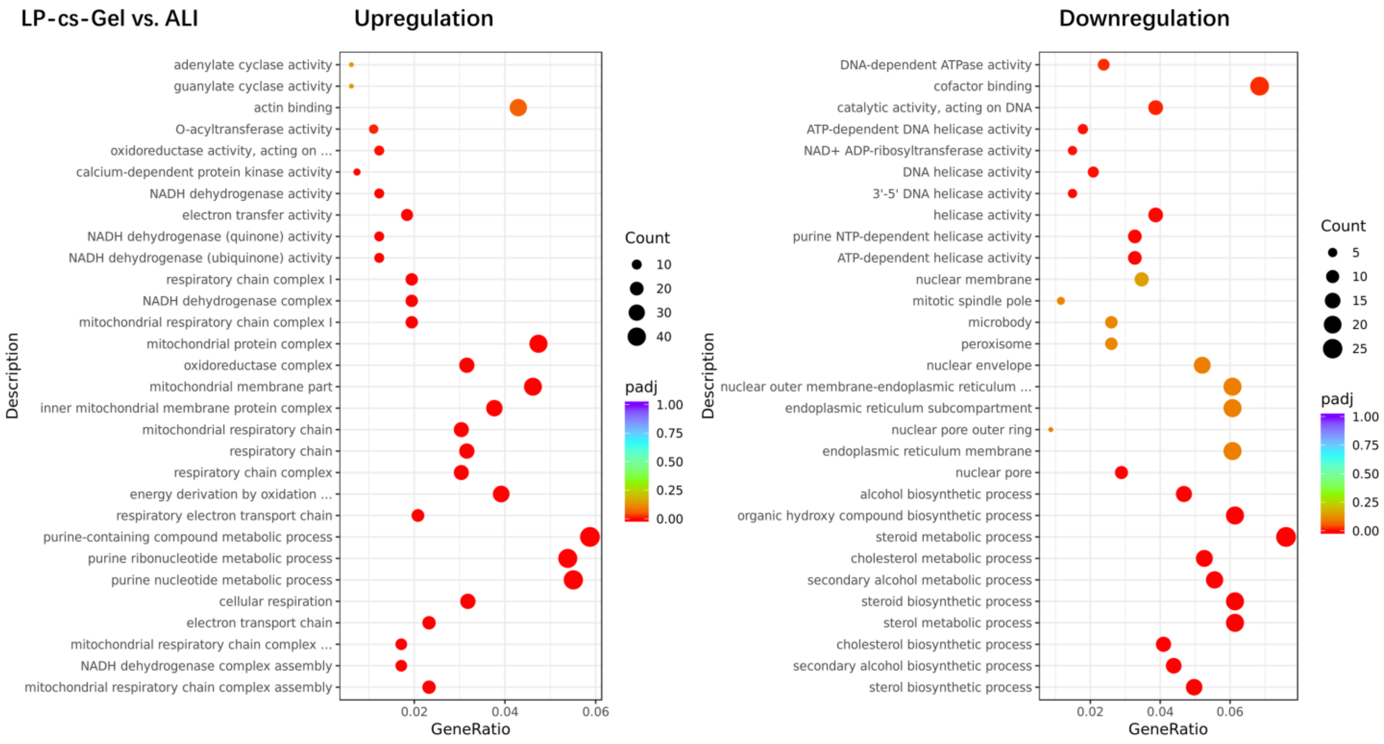

(C)

Figure S3. GO enrichments of the differentially expressed genes. A–C: GO enrichment results of (A): ALI vs. the control; (B) ALI vs. LP-cs-Gel; (C): ALI vs. LP-cs-Gel.

Table S1. The sequence of primers for amplifying the target genes.

Genes	Sequence (5'-3')	Length (bp)
GAPDH	F：AAGAAGGTGGTGAAGCAGG<br>R：GAAGGTGGAAGAGTGGGAGT	111
Sqle	F：TCTGGGAGATGCGTATAACCTG<br>R：TTCACAACAAAGGAATGGGTC	186
Cyp51	F：TGAGGAGCAAAACAGTAAGCAG<br>R：AAATCCCGAGGGTGAAGGT	152
Msmo1	F：ACTCACCACTTCATGCCATAGG<br>R：CAGGTTTCAGTCACTCGCTCA	194

Table S2. Mean consumption of daily water and dietary for 3 weeks in each group of mice.

Daily Consumption	Control (n=6)	ALI (n=6)	LP-cs (n=6)	LP-cs-Gel (n=6)
**Water (ml/day)**	5.76 ± 0.19	6.63 ± 0.28	6.55 ± 0.21	6.40 ± 0.39
**Diet (g/day)**	3.47 ± 0.13	3.67 ± 0.11	3.63 ± 0.12	3.13 ± 0.17
